# Allosteric Modulation of the HIV-1 gp120-gp41 Association Site by Adjacent gp120 Variable Region 1 (V1) N-Glycans Linked to Neutralization Sensitivity

**DOI:** 10.1371/journal.ppat.1003218

**Published:** 2013-04-04

**Authors:** Heidi E. Drummer, Melissa K. Hill, Anne L. Maerz, Stephanie Wood, Paul A. Ramsland, Johnson Mak, Pantelis Poumbourios

**Affiliations:** 1 Macfarlane Burnet Institute for Medical Research and Public Health, Melbourne, Victoria, Australia; 2 Department of Microbiology, Monash University, Clayton, Victoria, Australia; 3 Department of Microbiology and Immunology, The University of Melbourne, Melbourne, Victoria, Australia; 4 Department of Immunology, Monash University, Alfred Medical Research and Education Precinct, Melbourne, Victoria, Australia; 5 Department of Surgery, Austin Health, The University of Melbourne, Heidelberg, Victoria, Australia; 6 Department of Biochemistry and Molecular Biology, Monash University, Clayton, Victoria, Australia; 7 Deakin University School of Medicine, Geelong, Victoria, Australia; Harvard University, United States of America

## Abstract

The HIV-1 gp120-gp41 complex, which mediates viral fusion and cellular entry, undergoes rapid evolution within its external glycan shield to enable escape from neutralizing antibody (NAb). Understanding how conserved protein determinants retain functionality in the context of such evolution is important for their evaluation and exploitation as potential drug and/or vaccine targets. In this study, we examined how the conserved gp120-gp41 association site, formed by the N- and C-terminal segments of gp120 and the disulfide-bonded region (DSR) of gp41, adapts to glycan changes that are linked to neutralization sensitivity. To this end, a DSR mutant virus (K601D) with defective gp120-association was sequentially passaged in peripheral blood mononuclear cells to select suppressor mutations. We reasoned that the locations of suppressors point to structural elements that are functionally linked to the gp120-gp41 association site. In culture 1, gp120 association and viral replication was restored by loss of the conserved glycan at Asn^136^ in V1 (T138N mutation) in conjunction with the L494I substitution in C5 within the association site. In culture 2, replication was restored with deletion of the N^139^INN sequence, which ablates the overlapping Asn^141^-Asn^142^-Ser-Ser potential N-linked glycosylation sequons in V1, in conjunction with D601N in the DSR. The 136 and 142 glycan mutations appeared to exert their suppressive effects by altering the dependence of gp120-gp41 interactions on the DSR residues, Leu^593^, Trp^596^ and Lys^601^. The 136 and/or 142 glycan mutations increased the sensitivity of HIV-1 pseudovirions to the glycan-dependent NAbs 2G12 and PG16, and also pooled IgG obtained from HIV-1-infected individuals. Thus adjacent V1 glycans allosterically modulate the distal gp120-gp41 association site. We propose that this represents a mechanism for functional adaptation of the gp120-gp41 association site to an evolving glycan shield in a setting of NAb selection.

## Author Summary

The envelope glycoprotein gp120-gp41 complex of HIV-1 mediates receptor attachment and virus-cell membrane fusion, leading to cellular entry. A shield of asparagine-linked oligosaccharides occludes the gp120-gp41 protein surface and evolution of this glycan shield provides a means for evading circulating neutralizing antibody. Here we examined how conserved structural elements of the glycoprotein complex, in particular the gp120-gp41 association site, retain functionality in the context of glycan shield evolution. This information is important for the evaluation and exploitation of such conserved functional determinants as potential drug and/or vaccine targets. Our data indicate that the loss of either of 2 adjacent glycans in variable region 1 of gp120 leads to changes in local and remote glycan-dependent epitopes and that this is linked to a remodelling of gp120-gp41 interactions in order to maintain a functional gp120-gp41 complex. We propose that this represents a mechanism for the functional adaptation of the gp120-gp41 association site to an evolving glycan shield in a setting of neutralizing antibody selection.

## Introduction

**Figure 1:**
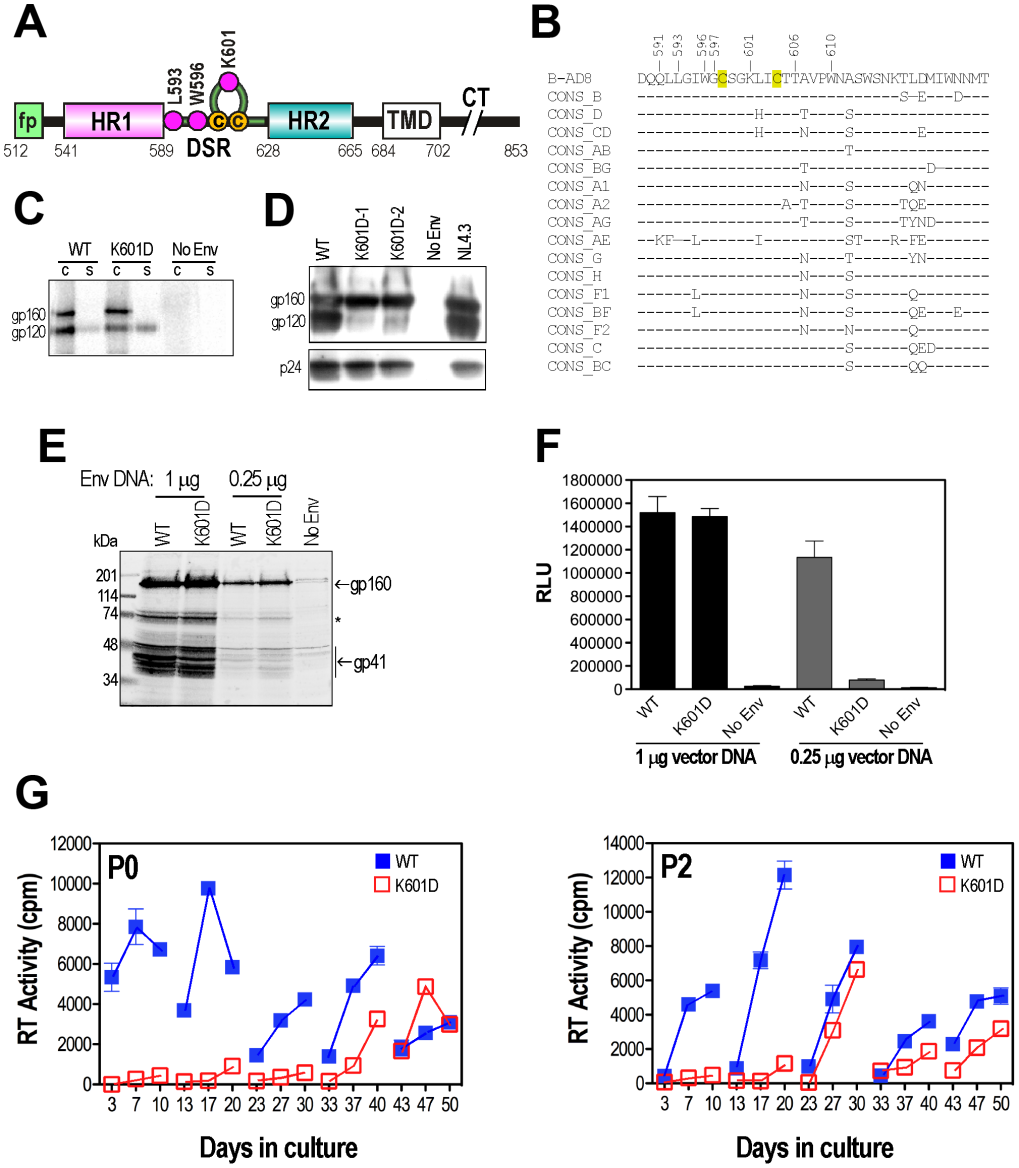
Location and phenotype of K601D. ***A***, Linear map of gp41. fp: fusion peptide, HR1: helical region 1, DSR: disulfide bonded region, HR2: helical region 2, TMD: transmembrane domain, CT: cytoplasmic tail. Residue numbering is in accordance with HXB2 Env. Magenta circles denote the residues mutated in this study. ***B***, Alignment of the AD8 DSR amino acid sequence with consensus (CONS) sequences derived from HIV-1 main-group subtypes and circulating recombinant forms (http://www.hiv.lanl.gov/content/sequence/NEWALIGN/align.html). The residue numbers of amino acids implicated in gp120 association [27], [76] are indicated. ***C***, gp120-gp41 association. Lysates of metabolically labelled WT, K601D or empty vector (mock) transfected 293T cells (c) and corresponding culture supernatants (s) were immunoprecipitated with IgG14 and protein G Sepharose. Proteins were analysed under reducing conditions in SDS-PAGE and scanned in a Fuji phosphorimager. Panel prepared from a single gel using Adobe Photoshop. ***D***, Virion characterization. HIV-1 virions produced by transfected 293T cells were pelleted from the culture supernatant through a 25% sucrose cushion and analysed by reducing SDS-PAGE and Western blotting with DV-012 (anti-gp120) and mAb 183 (anti-CA). K601D.1 and K601D.2 are 2 independent clones of pAD8-K601D; NL4.3: virus derived from the pNL4.3 clone [116]. ***E***, Lysates of 293T cells transfected with 1 or 0.25 µg pcDNA3.1-AD8*env* vectors analysed by Western blot using the gp41-specific mAb, C8. The asterisk denotes degradation products of gp160. ***F***, Cell-cell fusion. 293T effector cells were cotransfected with 1 µg pCAG-T7 plus 1 µg or 0.25 µg of pcDNA3.1-AD8*env* and then cocultured (16 h, 37°C) with BHK21 target cells that had been cotransfected with pc.CCR5 and pT4*luc*. The mean relative light units (RLU) ± standard deviation of a representative experiment is shown. ***G***, Long-term PBMC culture of HIV-1_AD8_-WT and HIV-1_AD8_-K601D. Viruses produced by pAD8-transfected 293T cells were normalized according to RT activity and then used to infect independent cultures of PHA stimulated PBMCs (cultures P0 and P2). The PBMCs used in each passage were obtained from different donors. Cell-free virus collected at day 10 of each passage was normalized for RT activity and used to infect fresh PHA stimulated PBMCs. The mean RT activity ± standard deviation of duplicate samples obtained from culture supernatants is shown.

The HIV-1 envelope glycoprotein (Env) complex comprises a trimer of gp120 subunits in non-covalent association with a trimer of transmembrane gp41 subunits and mediates viral attachment, membrane fusion and viral entry (for review see [1], [2]). Within gp120, 5 conserved regions (C1–C5) alternate with 5 variable regions (V1–V5). The conserved regions largely form the gp120 core comprised of inner and outer subdomains that are bridged by 4 antiparallel β-strands (the bridging sheet), whereas the variable regions form external solvent-exposed loops [3], [4], [5], [6], [7], [8]. gp120 is anchored to the viral envelope by the trimeric transmembrane/fusion glycoprotein, gp41. The ectodomain of gp41 comprises an N-terminal fusion peptide linked through N- and C-terminal α-helical heptad repeat sequences (HR1 and HR2, respectively) to a C-terminal membrane anchor and cytoplasmic tail. A central disulfide-bonded loop region or DSR joins HR1 to HR2 (**Fig. 1A, B**).

The membrane fusion and viral entry function of gp120-gp41 involves conformational changes that are triggered by receptors. CD4 ligation is believed to reorganize V1V2 and V3 to expose a binding site for the chemokine receptors CCR5 and CXCR4, which function as fusion cofactors [3], [4], [5], [6], [9], [10], [11], [12]. The V3 loop mediates important contacts with the negatively charged N-terminal domain and extracellular loop 2 of CCR5 and CXCR4 and determines the chemokine receptor preference of HIV-1 isolates. In a virion context, CD4 binding causes an “opening up” of the gp120 trimer due to outward rotation and displacement of gp120 monomers [10], [12].

gp120-receptor interactions cause gp41 to transition from a dormant metastable structure into a fusion active state [1], [2], [13], [14]. Structural transitions in gp41 that are associated with fusion function include the formation of a “prehairpin intermediate” structure wherein a triple-stranded coiled coil of HR1 segments provides a binding surface for the HR2, while studies with synthetic peptides and glycoprotein mutants indicate that the fusion peptide inserts into the target membrane [15], [16], [17], [18], [19]. Antiparallel HR1-HR2 interactions form a 6-helix bundle which apposes the N- and C-terminal membrane inserted ends of gp41, and the associated viral and cellular membranes, leading to merger and pore formation [20], [21], [22], [23], [24].

How conformational signals are transmitted between receptor-bound gp120 and gp41 to trigger the refolding of gp41 into the fusion-active state is being elucidated. A gp120-gp41 association site formed by the terminal segments of C1 and C5 of gp120 and the central DSR of gp41 [25], [26], [27], [28] may play an important role in this process as mutations in the DSR can inhibit CD4-triggered gp41 prehairpin formation and the initial hemifusion event [29]. Furthermore, the introduction of Cys residues to C5 and to the DSR generates an inactive disulfide-linked gp120-gp41 complex that is converted to a fusion-competent form by reduction [30], [31]. These findings implicate the C1–C5-DSR synapse in maintaining gp120-gp41 in the prefusion state and in subsequent transmission of fusion activation signals emanating from receptor-bound gp120. The terminal C1 and C5 gp41-contact regions project ∼35-Å from a 7-stranded β-sandwich at the base of the gp120 inner domain [8]. This β-sandwich appears to also play an important role in conformational signalling between gp120 and gp41 by linking CD4-induced structural changes in 3 structural layers of gp120 that emanate from the β-sandwich to the activation of gp41 [32].

Understanding how conserved functional determinants of the HIV-1 glycoproteins tolerate or adapt to the rapid evolution of other Env regions is important for their evaluation and exploitation as potential drug and/or vaccine targets. For example, resistance to a novel low molecular weight fusion inhibitor, PF-68742, is conferred by mutations in the DSR implicating this gp41 region as an inhibitor target [33]. Neutralizing antibodies exert strong evolutionary pressures on Env that can result in an increase in the number and/or a change in the position of potential N-linked glycosylation sites (PNGSs) that modify NAb-Env interactions [34], [35]. V1V2 is a key regulator of neutralization resistance, which generally correlates with its elongation and acquisition of PNGSs [34], [36], [37], [38], [39], [40], [41], [42], [43], [44]. Previously, we identified the C1–C5-DSR association site as a conserved determinant that exhibits structural and functional plasticity. This idea is based on the finding that whereas the gp120-gp41 association function and sequence of the DSR is conserved, the contribution of individual DSR residues to gp120 anchoring and membrane fusion function varies among HIV-1 strains and is controlled by V1V2 and V3 [28]. We proposed that this plasticity enables the maintenance of a functional glycoprotein complex in a setting of host selective pressures such as NAbs that drive the rapid coevolution of gp120 and gp41.

In the present study, we reveal a structural mechanism whereby the conserved gp120-gp41 association site adapts to changes in the glycan shield. Forced viral evolution was used to examine how the gp120-gp41 complex adapts to overcome a mutation (K601D in the DSR) that causes defective gp120-gp41 association. The defective phenotype was restored by second site mutations leading to the loss of PNGSs at V1 positions 136 and 142, the former operating in conjunction with L494I in C5 of gp120 and the latter requiring D601N in the DSR. The PNGS mutations appeared to exert their suppressive effects by altering the dependence of gp120-gp41 interactions on Leu^593^, Trp^596^ and Lys^601^ within the DSR. The PNGS mutations were also linked to an increase in the sensitivity of HIV-1 pseudovirions to neutralization by the glycan-directed NAbs 2G12 and PG16. Our data implicate V1 glycans linked to neutralization susceptibility in the allosteric modulation of the gp120-gp41 association site. We propose that this represents a mechanism for maintaining gp120-gp41 function in a setting of NAb-driven Env evolution.

## Results

### Phenotype and long-term culture of the HIV-1_AD8_-K601D DSR mutant

In this study, we subjected the gp120-gp41 association defective DSR mutant HIV-1_AD8_-K601D to serial PBMC passage in order to select suppressor mutations. We reasoned that the locations of suppressors would point to structural elements that are functionally linked to the DSR, thereby shedding light on its role in Env function. We first confirmed that the K601D mutation promoted the shedding of gp120 into the culture supernatant of 293T cells transfected with pcDNA3.1-AD8*env*, as determined by radioimmunoprecipitation (**Fig. 1C**). Consistent with this finding, K601D-mutated HIV-1_AD8_ virus particles were largely devoid of gp120 (**Fig. 1D**). We note the presence of gp160 in the pelleted virion preparations. It likely represents one of a number of non-native Env forms that have been observed in virions and have been implicated as immune decoys [45], [46], [47], [48], [49], [50]. The gp120 shedding defect did not appear to be associated with a precursor processing defect as Western blotting with the gp41-directed C8 monoclonal antibody (mAb), indicated that similar amounts of gp41 were derived from gp160 for both WT and K601D (**Fig. 1E**). Cell-to-cell fusion function was blocked by K601D at sub-saturating Env conditions (**Fig. 1F**), while HIV-1_AD8_ viral replication over 10 days in phytohemagglutinin-stimulated peripheral blood mononuclear cells (PBMCs) from independent donors was severely attenuated by the mutation (**Fig. 1G**). The restoration of HIV-1_AD8_-K601D replication competence was observed with sequential passage of cell-free virus in independent PHA-stimulated PBMC cultures (**Fig. 1G** **, cultures P0 and P2**).

### Genotypes of HIV-1_AD8_-K601D revertants

**Figure 2:**
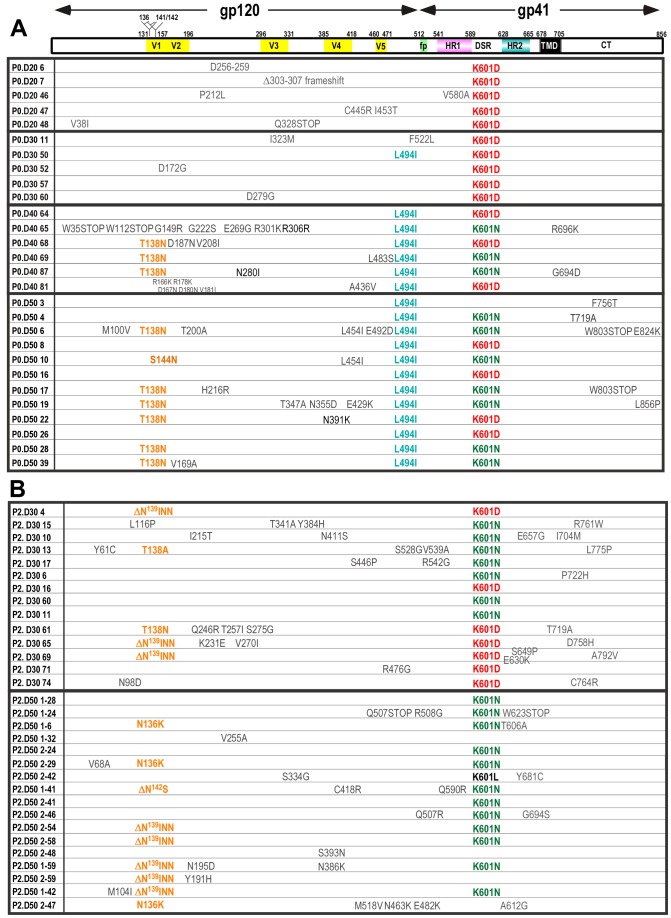
Genotypes of HIV-1_AD8_-K601D revertants. The *env* region of proviral DNA isolated at days 20, 30, 40 and 50 from P0 (***A***), and days 30 and 50 from P2 (***B***) was amplified by PCR and cloned into pGEM-T or pΔKAD8*env*. The entire *env* region present in individual clones was sequenced using Bigdye terminator 3.1. The amino acid numbering is based on HXB2 Env.

In order to identify suppressors of K601D, the *env* region was PCR-amplified from genomic DNA isolated from infected cells at days 20, 30, 40 and 50 for culture P0, and days 30 and 50 for culture P2. The PCR products were cloned into the pΔKAD8*env* expression vector for DNA sequencing. The K601D mutation was retained in P0 clones isolated at days 20 and 30 (FIG. 2A), when replication was not observed in the mutant virus culture (FIG. 1G, P0). The emergence of replication-competent virus at day 40 in the P0 culture correlated with the appearance of L494I in C5 of gp120 (6/6 clones), together with either a D601N pseudoreversion (3/6 clones), and/or T138N, which abolishes a conserved PNGS at position 136 (N^136^VT) within V1 of gp120 (3/6 clones). By day 50, when mutant viral replication approached that of WT, the T138N/L494I/K601N triple mutation was present in 42% of clones. A single clone (P0.D50.10) contained S144N, which ablates the nearby PNGS at N^142^SS.

In an independent PBMC long-term culture (P2), the replication competence of the mutant virus approached that of WT by the 3rd passage (**Fig. 1G** **, P2**). Deletion of residues N^139^INN (ΔN^139^INN) from V1, that results in the loss of overlapping PNGSs at positions 141 and 142 (N^141^N^142^SS), was observed in 3/14 P2 day-30 clones, while 2/14 possessed mutations at Thr^138^; K601N was also observed in 50% of P2-day 30 clones (**Fig. 2B**). The dominant genotypes in P2 day-50 clones were ΔN^139^INN together with K601N, and K601N alone. A 2-residue N^142^S^143^ deletion was observed in 1 day-50 clone (P2.D50.141), which also ablates the overlapping PNGSs at 141 and 142. Interestingly, the L494I mutation was not observed in P2 clones. These data indicate that PNGSs in V1 are functionally linked to the gp120-gp41 association synapse.

### Functional linkages between the DSR, PNGSs in V1, and Leu^494^ in C5

**Figure 3:**
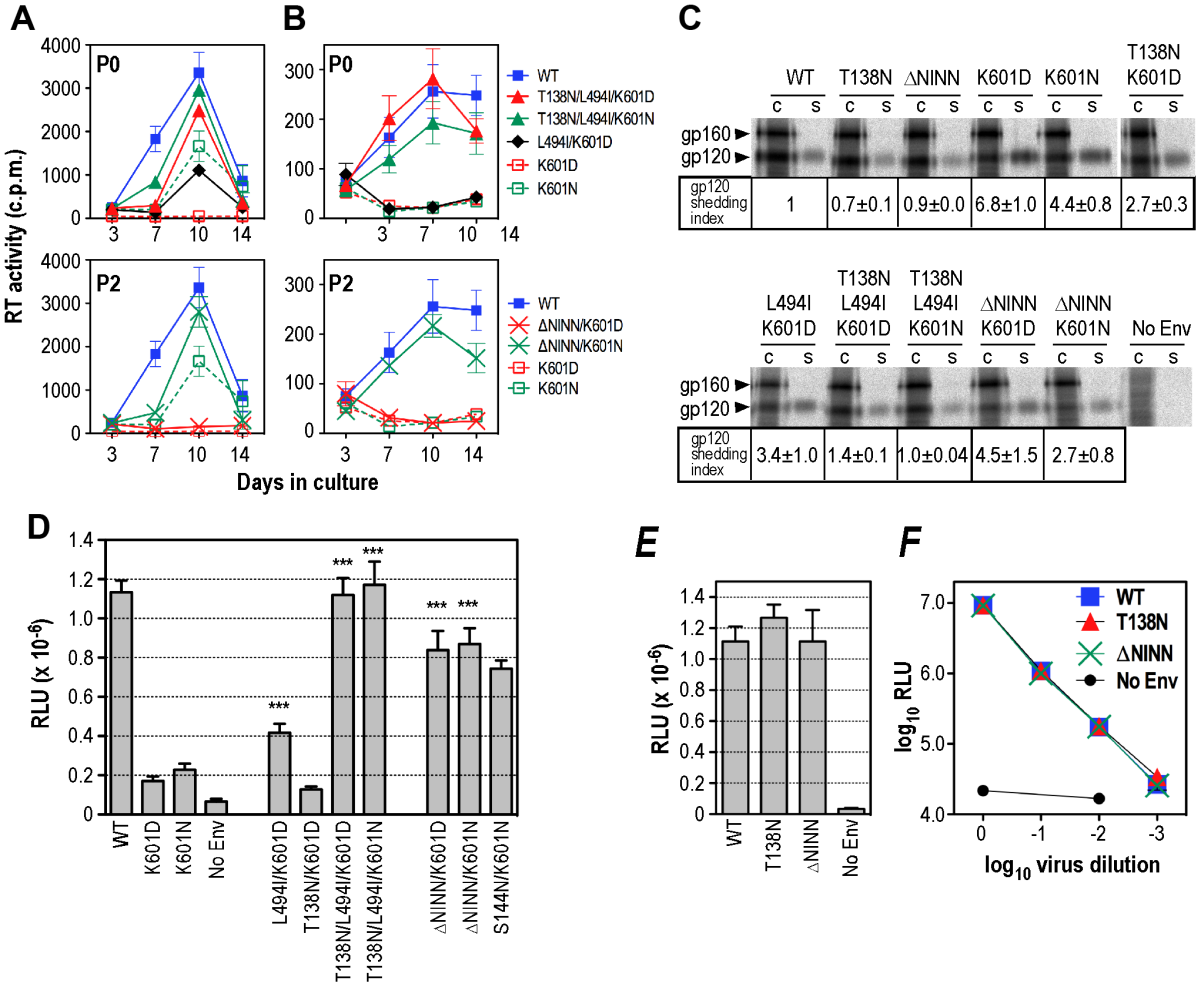
Analysis of revertants. ***A and B***, 14-day replication kinetics of representative P0 and P2 genotypes in PBMCs. Virus stocks produced in 293T cells were normalized according to RT activity and used to infect PHA stimulated PBMCs from 2 independent donors (***A*** **and** ***B*** **, respectively**). RT activity was measured in culture supernatants obtained at days 3, 7, 10 and 14 postinfection. The mean RT activity ± standard deviation of duplicate samples is shown. ***C***, gp120-gp41 association. Lysates of metabolically labelled Env-expressing cells (c) and corresponding culture supernatants (s) were immunoprecipitated with IgG14 plus protein G-Sepharose and subjected to reducing SDS-PAGE and phosphorimager scanning. The panel was prepared from 2 gels obtained from a single experiment (representative of 3 independent experiments) using Adobe Photoshop. gp120-shedding index was calculated according to the formula: ([mutant gp120]_supernatant_×[WT gp120]_cell_)/([mutant gp120]_cell_×[WT gp120]_supernatant_) [26]. The data shown are the mean shedding indices ± standard error from at least 3 independent experiments. ***D***, Cell-cell fusion activities of representative P0 and P2 genotypes. Assays were conducted with 0.25 µg AD8 Env expression plasmids as for FIG. 1*F*. Mean RLU ± standard error is shown (n>3). ***, *P*<0.001 *versus* K601D, 2-tailed unpaired t test assuming unequal variances. ***E***, T138N and ΔN^139^INN mutations on a WT Env background do not affect cell-cell fusion activity (Mean RLU ± standard error, n>3). ***F***, T138N and ΔN^139^INN mutations on a WT Env background do not affect the ability of Env-pseudotyped luciferase reporter viruses to mediate a single cycle of infection in U87.CD4.CCR5 cells. Mean RLU ± standard deviation from a representative experiment is shown.

To determine how replication was restored to the K601D virus in the long-term PBMC cultures, we reconstructed the dominant P0 and P2 genotypes in the context of the pAD8 proviral clone and examined viral replication in two independent PBMC donors. The P0 mutations, L494I within C5 and K601N within the DSR, partially restored replication in the PBMCs of one donor but not in the second, apparently less permissive donor (**Fig. 3A and B** **; P0 panels; Table S1**). The T138N/L494I/K601D and T138N/L494I/K601N genotypes exhibited near-WT replication kinetics in PBMCs from both donors. These data suggest that T138N, which ablates the PNGS at Asn^136^ within V1, and L494I act synergistically to suppress K601D (and K601N). The analysis of P2 genotypes in PBMC cultures indicated that the N^139^INN deletion in V1 was not sufficient to suppress K601D, but its combination with K601N led to near-WT levels of viral replication (**Fig. 3A, B** **; P2 panels**).

The pΔKAD8*env* expression vector was next employed to further dissect the functional linkages between position 601 within the DSR, the Asn^136^ and Asn^141/142^ PNGS mutations in V1 and the L494I mutation in C5 in gp120-gp41 association and cell-cell fusion assays. The results presented in **Fig. 3C** indicate that K601D and K601N DSR mutations promote shedding of gp120 into culture supernatants of transfected cells by ∼6.8- and 4.4-fold with respect to WT, respectively, while cell-cell fusion activity was reduced by ∼85–90% (**Fig.** **3D** **; Table S1**). These data indicate that the K601D and K601N DSR mutations inhibit cell-cell fusion function by destabilizing the gp120-gp41 complex. However, the effect of K601N on Env function in the context of virus replication appears to be donor dependent (**see** **Fig. 3A and B**). It may be that lower receptor numbers and/or alternate coreceptor post-translational modifications on target cells, or differences in target cell population numbers [51], [52], [53], [54] in the case of the latter donor's PBMCs do not enable the fusion-activation threshold to be reached for K601N Env. A general correlation between the restoration of cell-cell fusion activity and gp120-gp41 association was observed for the P0 combination mutants. Thus L494I/K601D exhibited ∼50% of WT cell-cell fusion activity with improved gp120-gp41 association, whereas T138N/L494I/K601D and T138N/L494I/K601N were functionally similar to the WT. The phenotype of T138N/K601D was an outlier as gp120-gp41 association was improved by ∼2-fold with respect to K601D without a restorative effect on Env fusion function. These data indicate that T138N and L494I act cooperatively to restore gp120-gp41 association to K601D with concomitant restoration of membrane fusion function and viral replication competence. T138N-containing gp120 molecules migrated to lower molecular weight positions with respect to WT in reducing SDS-PAGE, consistent with loss of the glycan at Asn^136^ (**Fig. 3C**).

The ΔN^139^INN/K601D and ΔN^139^INN/K601N P2 clones exhibited 74–77% of WT fusion activity (*P*<0.05) with only partial restoration of gp120-gp41 association, even though only the latter clone was competent for replication in PBMCs (**Figs. 3A–D** **, Table S1**). The suppression of the K601N fusion and replication phenotype by ΔN^139^INN is therefore not dependent on the full restoration of gp120-gp41 association or on the L494I C5 mutation. gp120 molecules with the ΔN^139^INN mutation migrated faster in SDS-PAGE than non-ΔN^139^INN-containing Envs, again consistent with the loss of glycan (**Fig. 3C**). The ΔN^139^INN mutation disrupts the overlapping N-linked glycosylation sequons: Asn^141^-Asn^142^-Ser-Ser. Such overlapping N-linked glycosylation sequons are observed in V1 of a subset of HIV-1 strains (http://www.hiv.lanl.gov/content/sequence/NEWALIGN/align.html#comp), although the position varies. Combining S144N (a PNGS mutation observed in clone P0.D50.10) with K601N resulted in almost identical fusion activity to ΔN^139^INN/K601N (**Fig. 3D**), suggesting that loss of the Asn^142^ glycan can substitute for the N^139^INN deletion.

Scanlan and coworkers [55], [56] reported that gp120 derived from virions produced in 293T cells and PBMCs is composed of oligomannose type glycans, whereas monomeric gp120 produced in 293T cells contains predominantly complex type glycans in addition to the oligomannose type. It was therefore important to determine whether the presence of complex type glycans on cell-surface expressed Env was influencing the results of the cell-cell fusion and gp120-gp41 association assays. Kifunensine, an α-mannosidase I inhibitor that blocks the synthesis of Man_5_GlcNac_2_, the precursor of hybrid and complex-type glycans [57], was used to restrict Env glycosylation to the oligomannose type. The culturing of Env-expressing 293T cells in increasing concentrations of kifunensine did not appear to affect the efficiency of gp160 processing to gp120 and gp41, however, the resultant gp120 migrated faster than its counterpart expressed in the absence of kifunensine. Furthermore, the gp120 and gp41 bands had a more focused appearance relative to the corresponding glycoproteins expressed under control conditions (**Fig. S1** ***A***). These data are consistent with homogeneous oligomannose type Env glycosylation due to the action of kifunensine [58]. We next examined the effects of 10 µM kifunensine on the cell-cell fusion activities of the T138N/L494I/K601N and ΔN^139^INN/K601N revertant Envs. **Figure S1** ***B*** shows that the presence of kifunensine leads to a general elevation in the fusion activities of the 4 constructs tested. Importantly, the presence of kifunensine did not affect the relative fusion activities of the T138N/L494I/K601N and ΔN^139^INN/K601N revertant Envs with respect to wild type and K601D. These data were reflected in the gp120-gp41 association assay where the shedding phenotype of K601D was restored to wild type and near-wild type levels, respectively, in T138N/L494I/K601N and ΔN^139^INN/K601N, in both the presence and absence of kifunensine (**Fig. S1** ***C***). Overall, these data indicate that the fusion and gp120-gp41 association phenotypes of the cell-expressed revertant Envs are not influenced by the presence of complex or hybrid-type glycans.

We next asked whether the V1 PNGS mutations restored function via a functional link to Asp or Asn at position 601 in the DSR or whether a generalized enhancement in Env function could explain the restored phenotypes. **Figures 3E and F** indicate that T138N and ΔN^139^INN do not increase the cell-cell fusion activity of surface expressed Env or the infectivity of Env-pseudotyped luciferase reporter virus when introduced to the WT background. We also noted that the L494I mutation in a WT background did not substantially increase cell-cell fusion or gp120-gp41 association (data not shown). These data are consistent with specific functional crosstalk between the V1 glycans, the Asp^601^ or Asn^601^ in the DSR and position 494 in C5.

**Figure 4:**
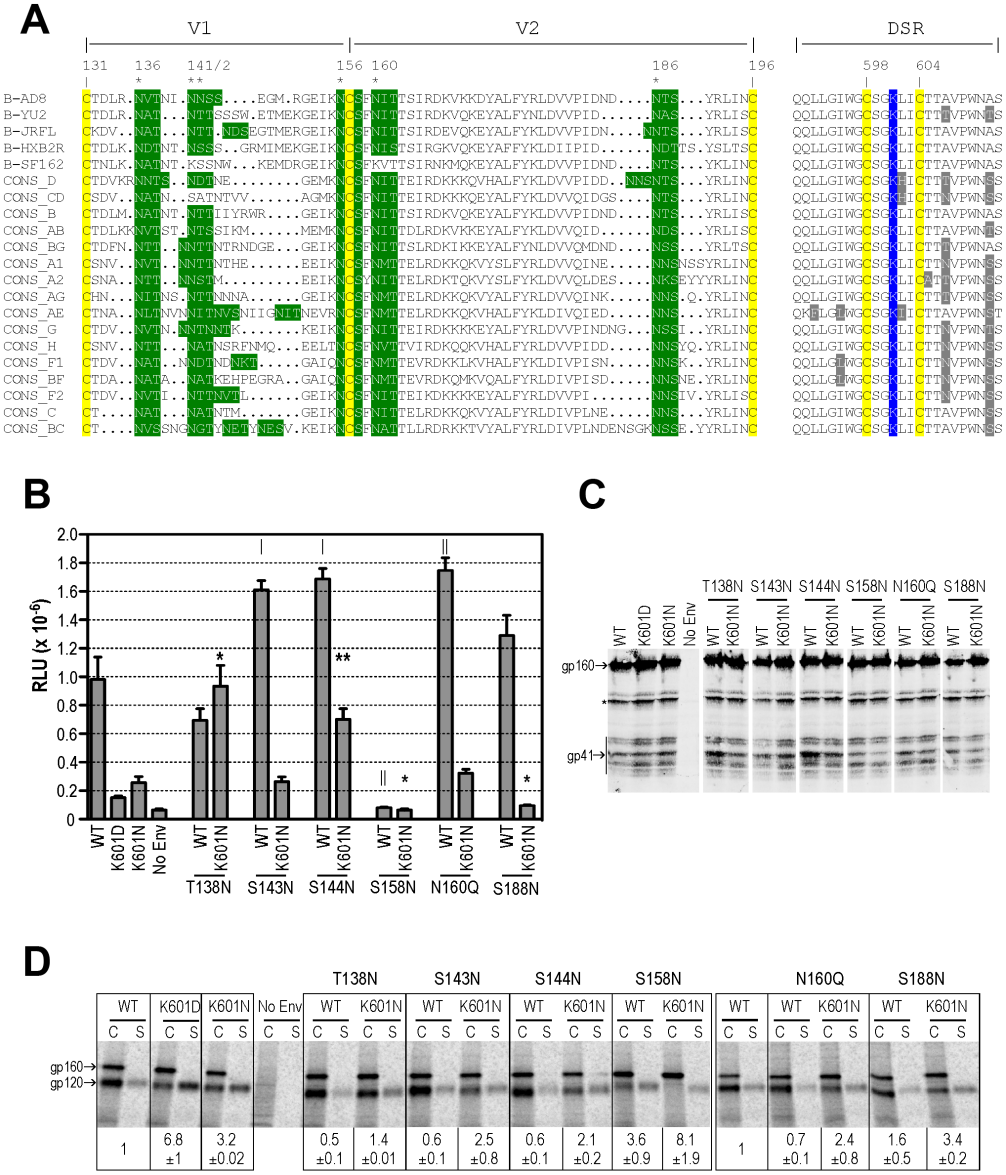
Evidence for a specific functional linkage between position 601 of the DSR and the Asn^136^ and Asn^142^ glycans of V1. ***A***, Alignment of V1V2 and corresponding DSR sequences. PNGSs are highlighted in green and numbered according to HXB2 Env. Variable residues in the DSR are highlighted in grey. CONS, subtype consensus sequence. ***B***, Cell-cell fusion. Assays were conducted with 0.25 µg Env expression plasmids as for FIG. 1*F*. Mean RLU ± standard error (n≥4) is shown. |, *P*<0.05; ‖, P<0.01 versus WT; *, *P*<0.05; ** *P*<0.01 *versus* K601N; 2-tailed unpaired t test assuming unequal variances. ***C***, Lysates of Env-expressing 293T cells were analysed by Western blot using the gp41-specific mAb, C8. The asterisk denotes degradation products of gp160. ***D***, gp120-gp41 association. Lysates of metabolically labelled Env-expressing cells (c) and corresponding culture supernatants (s) were immunoprecipitated with HIVIG plus protein G-Sepharose and subjected to reducing SDS-PAGE and phosphorimager scanning. The data are representative of 2 independent experiments. gp120-shedding indices (mean ± standard error) are shown below the corresponding immunoprecipitations and were calculated as for **FIG. 3** ***C*** from 2 independent experiments.

Finally, we asked if the V1 PNGS mutations at 136 and 142 restored function via a specific link to position 601 in the DSR, or whether deletion of any of the 6 PNGSs in V1V2 of the AD8 strain (**Fig. 4A**) could restore functional defects related to a DSR mutation. To this end, we introduced T138N, S143N, S144N, S158N, N160Q and S188N mutations to the WT and K601N pΔKAD*env* vectors and determined their effects on cell-cell fusion, glycoprotein processing and gp120-gp41 association. **Figure 4B** indicates that in a WT Env context, T138N and S188N did not affect cell-cell fusion, S143N, S144N and N160Q led to small but significant functional enhancements, whereas S158N blocked fusion completely. Even though, most of the V1V2 glycan mutants were fusogenic on a WT background, only T138N and S144N restored function to K601N. A Western blot of ΔKAD*env*-transfected 293T cell lysates with the gp41-specific mAb, C8, confirmed that the mutant glycoproteins were expressed and processed to gp41 at levels that were close to the WT (**Fig. 4C**). Pulse-chase biosynthetic labeling followed by radioimmunoprecipitation with pooled IgG derived from HIV-1-infected individuals (HIVIG) indicated WT-like gp120-gp41 association for T138N, S143N, S144N, N160Q and S188N and a shedding phenotype for the fusion-defective S158N (**Fig. 4D**). Near-WT levels of gp120-gp41 association were observed for the fusion-competent T138N/K601N mutant, whereas S144N/K601N, which is also fusion competent, exhibited a mild gp120 shedding phenotype. By contrast, improvements in gp120-gp41 association did not follow the combination of K601N with S143N, S158N, N160Q or S188N, consistent with their lack of fusion activity. Overall, these data are consistent with a specific functional linkage between the DSR and the Asn^136^ and Asn^142^ glycosylation sites in V1.

### Mechanism of reversion

**Figure 5:**
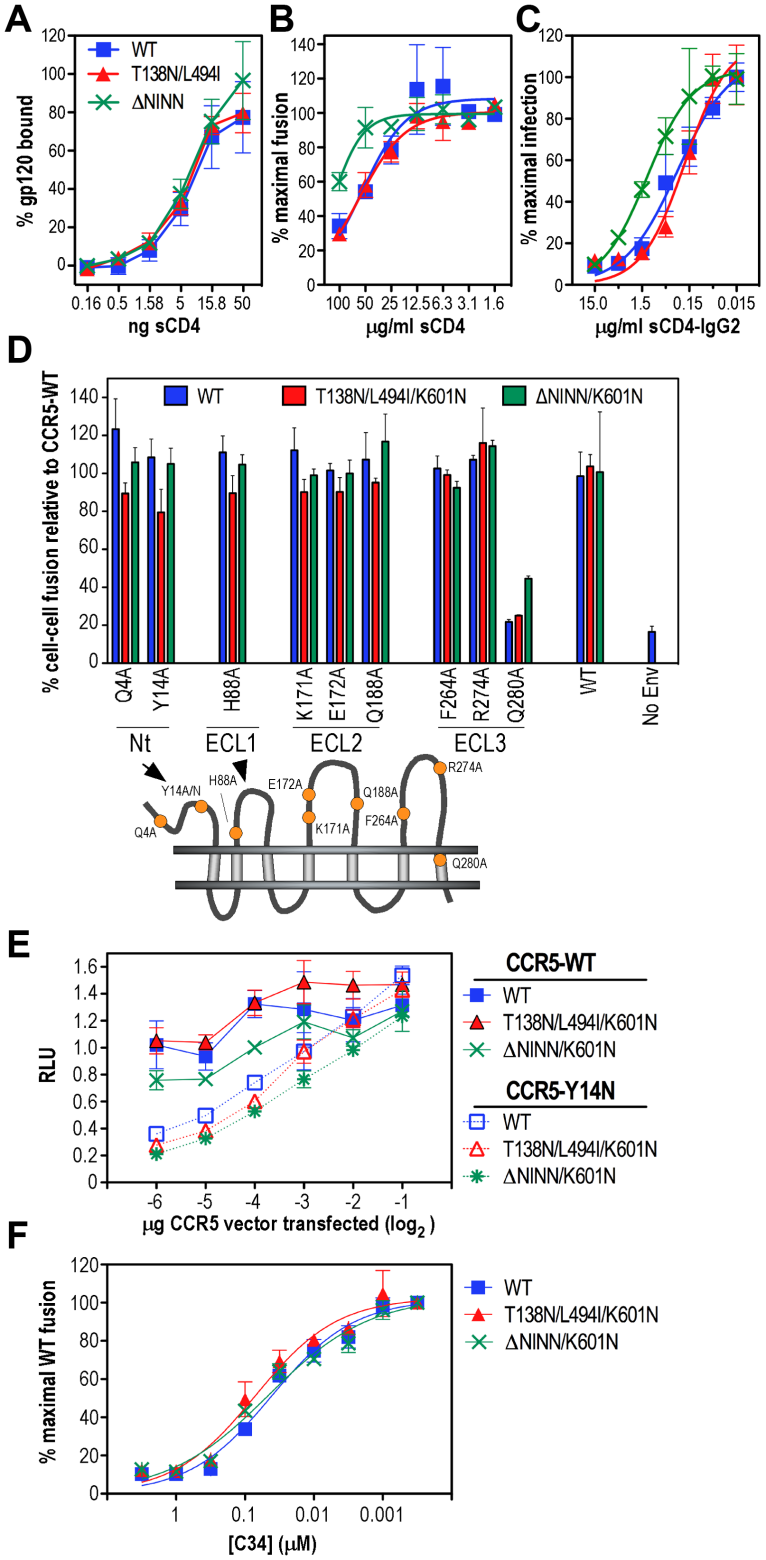
Receptor binding properties and C34 susceptibility of revertants. ***A***, CD4 binding by WT and mutated gp120 molecules. Soluble CD4 binding curves were obtained by incubating a constant amount of biosynthetically labelled WT and mutated gp120 with the indicated amounts of sCD4. gp120-sCD4 complexes were coimmunoprecipitated with mAb OKT4 and protein G-Sepharose, followed by reducing SDS-PAGE and densitometry of gp120 bands. gp120-sCD4 binding is expressed as a percentage of gp120 immunoprecipitated by IgG14. The mean ± standard deviation from 2 independent experiments is shown. ***B***, Inhibition of cell-cell fusion by sCD4. Assays were conducted with 0.25 µg Env expression plasmids as for **Fig. 1** ***F*** except that Env-293T cells were incubated with a dilution series of sCD4 for 3.5 h prior to coculture with CD4-plus-CCR5-expressing BHK21 targets for 8 h. The data are expressed as a percentage of the maximal fusion achieved by each Env and represent the means ± standard error from 4 independent experiments. WT: blue square; T138N/L494I/K601N: red triangle; ΔN^139^INN/K601N: green “X”. ***C***, Inhibition of infection by sCD4-IgG2. WT, T138N/L494I/K601N and ΔN^139^INN/K601N viruses were incubated with sCD4-IgG2 for 4 h at 37°C and then added to TZM-bl cells. The cells were lysed 48 h after infection and the luciferase activity present in the lysates determined. The data are expressed as a percentage of the maximal infection achieved by each virus and represent the means ± standard deviation from a single experiment performed in triplicate. Symbols are as for **Fig. 5** ***B***. ***D***, Utilization of CCR5 mutants in cell-cell fusion by T138N/L494I/K601N and ΔN^139^INN/K601N. Assays were conducted with 0.25 µg Env expression plasmids as for **Fig. 1** ***F***. The fusion activity of each Env clone was normalized against its fusion activity with WT CCR5. Mean relative fusion activity ± standard deviation is shown from a representative experiment. CCR5 N-terminal domain: Nt, extracellular loops 1, 2, and 3: ECL1, ECL2 and ECL3, respectively. ***E***, Utilization of the CCR5-Y14N coreceptor mutant in cell-cell fusion by T138N/L494I/K601N and ΔN^139^INN/K601N. 293T effector cells cotransfected with 1 µg pCAG-T7 plus 0.25 µg Env expression plasmids were cocultured (16 h, 37°C) with BHK21 targets cotransfected with 1 µg pT4*luc* plus the indicated amounts of WT or Y14N-mutated pc.CCR5 prior to luciferase assay. The fusion activity of each Env clone was normalized against its fusion activity with WT CCR5. Mean relative fusion activity ± standard deviation is shown from a representative experiment. ***F***, Sensitivity of T138N/L494I/K601N and ΔN^139^INN/K601N to the fusion inhibitor peptide, C34. 293T effector cells cotransfected with 1 µg pCAG-T7 plus 0.25 µg Env expression plasmids were cocultured (16 h, 37°C) with BHK21 targets cotransfected with pT4*luc* plus pc.CCR5 in the presence of the indicated amounts of C34 prior to luciferase assay. The data are expressed as a percentage of cell-cell fusion activity in the absence of inhibitor (mean ± standard error; n = 3).

The effects of reversion-associated mutations on gp120-CD4 binding, sensitivity of cell-cell fusion and infectivity to sCD4 inhibition, CCR5 utilization and sensitivity to the HR2-based fusion inhibitor peptide, C34, were next examined in order to further elucidate the mechanism whereby function was restored in the context of a mutated DSR. CD4 binding curves were generated by incubating a constant amount of biosynthetically labelled WT and mutated monomeric gp120 with serial dilutions of sCD4, coimmunoprecipitation of gp120-sCD4 complexes with mAb OKT4, followed by SDS-PAGE and densitometry of gp120 bands. Similar CD4 binding curves were observed for WT, T138N/L494I and ΔN^139^INN gp120 mutants (**Fig. 5A**). The sCD4 EC_50_ for WT, T138N/L494I and ΔN^139^INN gp120 was ∼0.5 nM, which approximates the published affinity range of sCD4 for monomeric gp120 [59]. These data indicate that the glycosylation site mutations in V1 and L494I in C5 did not alter the CD4-binding ability of monomeric gp120. We next compared the sensitivity of T138N/L494I/K601N-, ΔN^139^INN/K601N- and WT-Env-mediated fusion to inhibition with sCD4. The Env-expressing 293T cells were incubated with a sCD4 dilution series for 3.5 h prior to an 8-h coculture with CD4-plus-CCR5-expressing BHK21 targets harboring a luciferase reporter. **Figure 5B** indicates that WT and T138N/L494I/K601N have almost identical sensitivities to sCD4 with IC_50_s of ∼60 µg/ml, which are comparable to previously published values for both T cell-line adapted and primary HIV-1 Envs [60]. The ΔN^139^INN/K601N inhibition curve, however, was shifted by ∼1log_2_ to the left, indicating that this Env is slightly more resistant to sCD4, even though monomeric gp120 containing the ΔN^139^INN mutation had similar sCD4 binding characteristics to WT and T138N/L494I. We sought to recapitulate the cell-cell fusion data in a virus infection system. For this, we used TZM-bl luciferase reporter cells and the CD4-IgG2 fusion protein wherein the Fv regions of IgG have been replaced by domains 1 and 2 of CD4. The tetrameric nature of CD4-IgG2 leads to a reduction in IC_50_ relative to monomeric sCD4 [60]. Complete inhibition of virus infectivity was achieved with 15 µg/ml of CD4-IgG2 with WT and T138N/L494I/K601N exhibiting similar sensitivities to the fusion protein (IC_50_∼0.3 µg/ml) (**Fig. 5C**). As was observed in the cell-cell fusion assay, the ΔN^139^INN/K601N virus inhibition curve was shifted to the left, with an IC_50_ of ∼1.3 µg/ml, confirming that ΔN^139^INN/K601N is more resistant to the CD4-based inhibitor relative to WT and T138N/L494I/K601N in a virion context.

The abilities of T138N/L494I/K601N, ΔN^139^INN/K601N and WT Envs to utilize a panel of CCR5 coreceptor mutants were next compared in order to determine if alterations to the mode of CCR5 engagement could account for reversion. The results presented in **Fig. 5D** indicate that the T138N/L494I/K601N, ΔN^139^INN/K601N and WT Envs exhibited almost identical patterns of mutant CCR5 utilization. For example, Q4A and Y14A (N-terminal domain, Nt), H88A (extracellular loop 1, ECL1), K171A, E172A and Q188A (extracellular loop 2, ECL2), and F264A and R274A (extracellular loop 3, ECL3) CCR5 mutants supported fusion with the 3 Env constructs to the same extent as WT CCR5, whereas fusion with Q280A (extracellular loop 3) was decreased to 25–40% of WT CCR5 activity. We also compared the abilities of T138N/L494I/K601N, ΔN^139^INN/K601N and WT Envs to mediate cell-cell fusion with the CCR5-Y14N tyrosine sulfation mutant, which exhibits a lower affinity for gp120-gp41 and functions as a HIV-1 coreceptor in a cell surface concentration-dependent manner [61]. The coreceptor activity of WT CCR5 for WT and T138N/L494I/K601N remained at consistently high levels for fusion with BHK21 targets cotransfected with a constant amount of pT4*luc* vector and a dilution series of pc.CCR5 DNA; the fusion activity of ΔN^139^INN/K601N was slightly diminished across the pc.CCR5 dilution series (**Fig. 5E**), consistent with the data presented in **Fig. 3D**. These data are consistent with previous findings indicating that trace amounts of CCR5 are sufficient to mediate efficient fusion and entry by HIV-1 [61]. By contrast, fusion mediated by the 3 Env constructs exhibited a strict dependence on the amount of transfected CCR5-Y14N plasmid without any significant changes in the CCR5-Y14N concentration curves due to the Env mutations being observed. These data indicate that the restoration of function to the mutated DSR by the T138N/L494I or ΔN^139^INN suppressor mutations in gp120 is unlikely to be a result of altered CD4 and CCR5 utilization.

We considered that an increase in the efficiency of a post receptor-binding event, such as 6-helix bundle formation, could aid in the functional compensation of the DSR mutations. We therefore asked if the mutations in gp120 and K601N led to changes in sensitivity to the HR2 synthetic peptide analogue, C34, which blocks fusion by binding to the coiled coil of HR1 helices in a receptor-triggered prehairpin intermediate conformation of gp41 [17], [62]. We expect that faster 6-helix bundle folding kinetics would correspond to decreased C34 sensitivity. **Figure 5F** indicates that T138N/L494I/K601N, ΔN^139^INN/K601N and WT Envs exhibited almost identical C34 fusion inhibition curves with IC_50_s of ∼100 nM. These data indicate that the T138N/L494I/K601N reversion mechanism is unlikely to be due to changes in gp120-receptor interactions nor post-receptor binding events such as efficiency of 6-helix bundle formation, whereas ΔN^139^INN/K601N may be subtly altered in sCD4-induced changes that inhibit membrane fusion function.

### Modulation of the gp120-gp41 association site by adjacent glycosylation sites in V1

**Figure 6:**
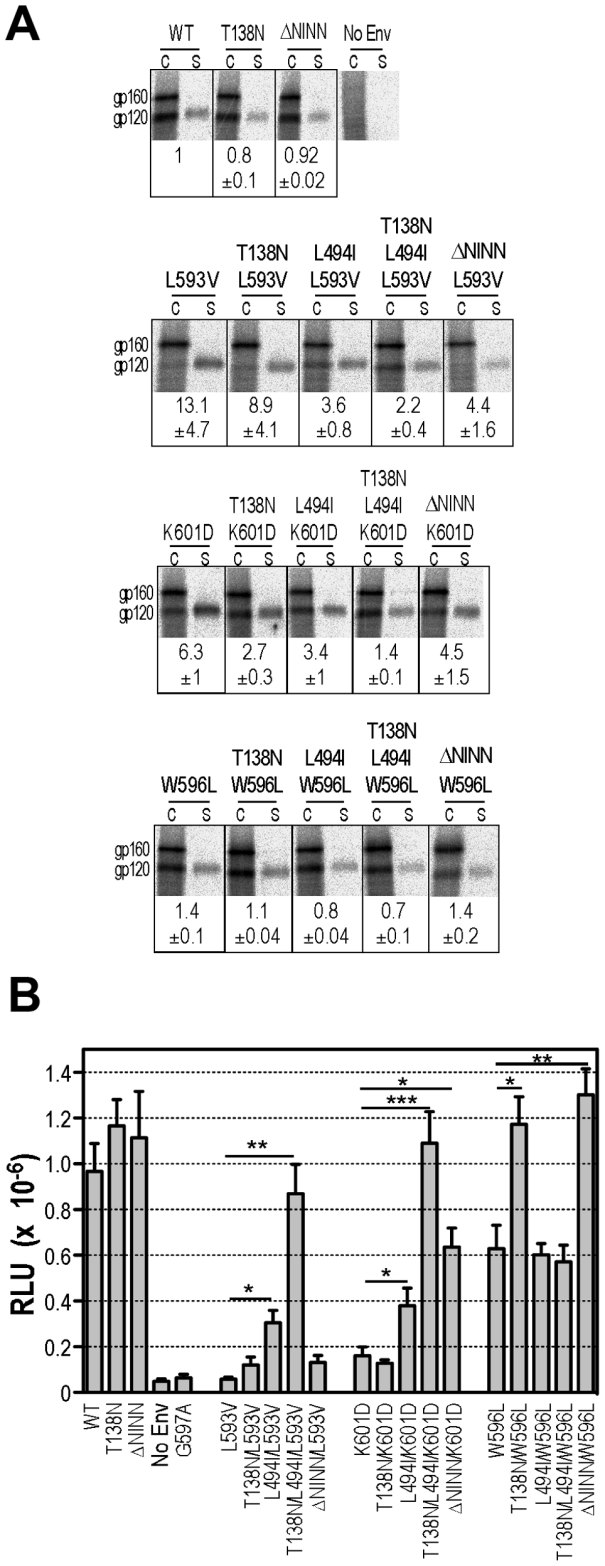
Effects of T138N, ΔN^139^INN and L494I mutations on gp120-gp41 association and cell-cell fusion activities of DSR mutants. ***A***, gp120-gp41 association. Lysates of metabolically labelled Env-expressing cells (c) and corresponding culture supernatants (s) were immunoprecipitated with IgG14 plus protein G-Sepharose and subjected to SDS-PAGE and phosphorimager scanning. The panel was prepared from 3 gels obtained from a single experiment using Adobe Photoshop. gp120-shedding indices (mean ± standard error) are shown below the corresponding immunoprecipitations and were calculated as for **Fig. 3** ***C*** from at least 3 independent experiments. ***B***, Cell-cell fusion. Assays were conducted with 0.25 µg Env expression plasmids as for FIG. 1*F*. Mean RLU ± standard error (n≥3) is shown. *, *P*<0.05; ** *P*<0.01; **, *P*<0.001; 2-tailed unpaired t test assuming unequal variances.

The DSR mediates association with gp120 and may play a role in the activation of gp41 by responding to receptor-induced changes in gp120 [27], [28], [29], [30], [31]. To better understand how glycosylation in V1 impacts on gp120-gp41 interactions, we assessed the functional effects of T138N, L494I, T138N/L494I and ΔN^139^INN mutations on two other gp120 contact residues within the DSR, Leu^593^ and Trp^596^, in addition to Lys^601^ [27], [28]. While L593V and K601D mutations resulted in decreased gp120-gp41 association and cell-cell fusion function, the W596L mutant exhibited WT levels of gp120-gp41 association but reduced cell-cell fusion by ∼40% at subsaturating Env (*P*<0.02, 2 sample t-test, unequal variances) (**Fig. 6A, B** **).** **Figures 6A and B** indicate that T138N had no effect on cell-cell fusion when combined with L593V or K601D, whereas small improvements in glycoprotein association and fusion function were observed when L494I was added to the DSR mutants; wild type levels of gp120-gp41 association and fusion were attained when both T138N and L494I were combined with L593V or K601D. By contrast, W596L exhibited WT fusion levels in combination with T138N. The N^139^INN deletion did not provide any improvement to L593V fusogenicity, but restored increasing levels of fusion function to K601D and W596L, respectively. These data indicate that T138N in V1 alters the gp120-gp41 association site such that fusion function is less dependent on Trp^596^ and, when L494I is also present, on Leu^593^ and Lys^601^. The N^139^INN deletion renders fusion function less dependent on Trp^596^ and Lys^601^.

### Modulation of neutralization sensitivity by adjacent glycans in V1

**Figure 7:**
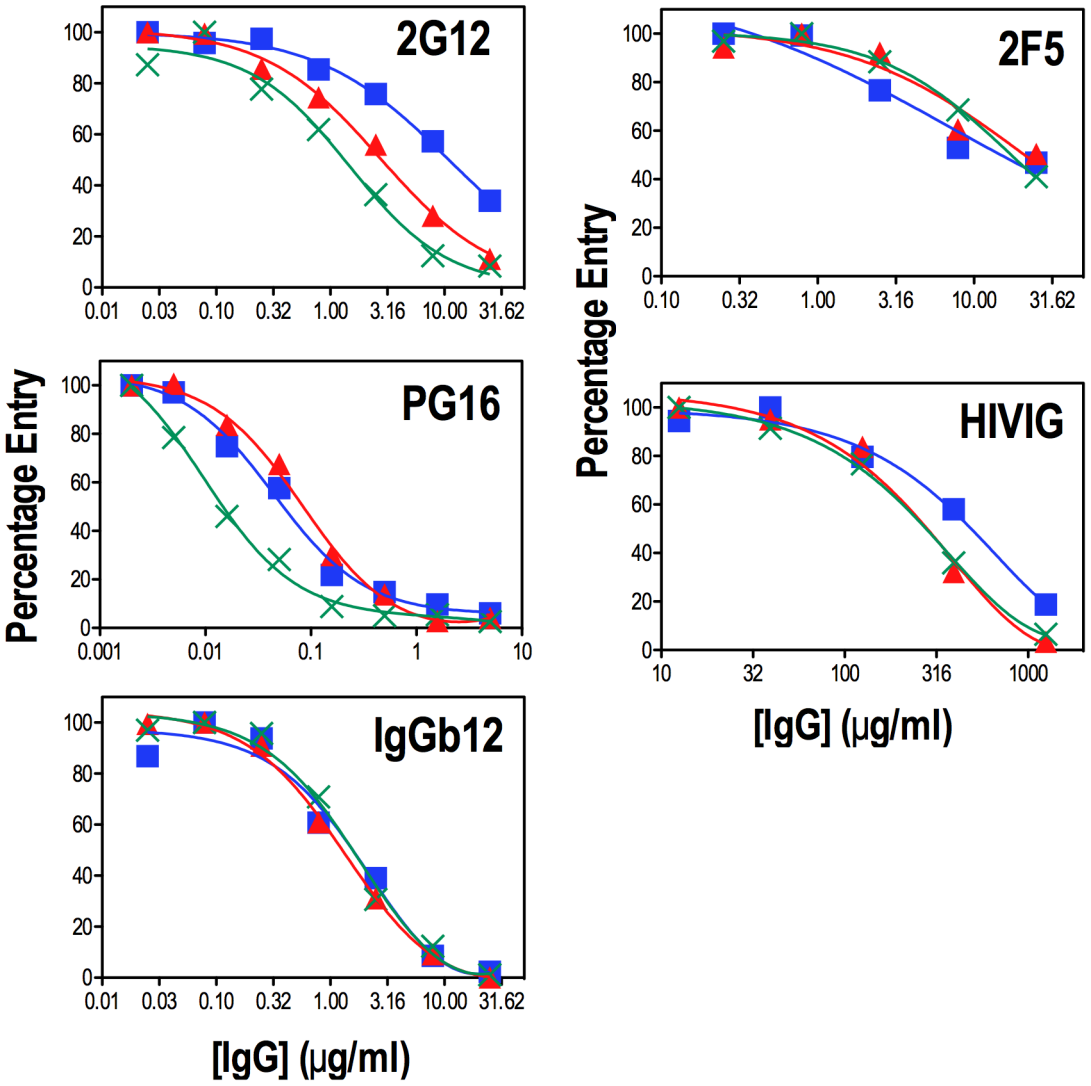
Sensitivity of T138N and ΔN^139^INN mutant pseudovirions to NAbs. U87.CD4.CCR5 cells were incubated with pseudovirus-IgG mixtures for 2 days prior to lysis and assay for luciferase activity. Neutralizing activities were measured in triplicate and reported as the average percent luciferase activity. The data are representative of 2–4 independent experiments. WT: blue squares, T138N: red triangles, ΔN^139^INN: green “X”s.

Previous studies have shown that larger numbers of potential N-linked glycans in V1V2 correlate with resistance to NAb [34], [37], [38], [39], [40], [42]. We therefore asked whether the T138N and ΔN^139^INN mutations were linked to changes in the neutralization sensitivity of Env-pseudotyped reporter viruses. The results (**Fig. 7**) show step-wise ∼0.5log_10_-increases in the neutralization sensitivities of T138N and ΔN^139^INN to the monoclonal NAb 2G12, which is directed to a glycan cluster involving Asn^295^, Asn^332^ Asn^339^, Asn^386^ and Asn^392^ on the outer face of gp120 [63], [64], [65], [66], [67], [68]). The 2G12 IC_50_s for WT, T138N and ΔN^139^INN were 13, 4 and 1.5 µg/ml, respectively. In the case of PG16, which recognizes an epitope in V1V2 involving the Asn^156^ and Asn^160^ oligomannose glycans [7], [69], [70], neutralization was enhanced for ΔN^139^INN only (IC_50_ = 0.075 and 0.015; IC_90_ = 1.6 and 0.16 µg/ml, respectively, for WT and ΔN^139^INN). By contrast, neutralization by IgGb12 (CD4 binding site [71], [72]) and 2F5 (membrane proximal external region of gp41 [73], [74], [75]) was not affected by the V1 mutations. Pooled IgG from HIV-1 infected individuals (HIVIG) was used as a reference reagent. In this case, a reproducible ∼2-fold increase in sensitivity to neutralization by HIVIG was observed with the V1 PNGS mutations (IC_50_∼550 µg/ml for WT, 280 µg/ml for T138N and ΔN^139^INN) indicating that these glycans are likely to modulate neutralization epitopes recognized by human immune sera. These data indicate that the adjacent V1 glycans shown here to modulate the gp120-gp41 association site are linked to neutralization resistance.

## Discussion

The gp120-gp41 complex of HIV-1 is maintained through interactions between C1 and C5 of gp120 with the DSR of gp41. Receptor engagement elicits a conformational signal that is transmitted through gp120 and sensed by the gp120-contact residues to activate the membrane fusion potential of gp41. In this study, we forced the evolution of a DSR mutant (K601D) with defective gp120-gp41 association, reasoning that the locations of emergent 2^nd^ site mutations that suppress the defect will point to structural elements within gp120-gp41 with functional links to the DSR. The loss of glycans at Asn^136^ or Asn^142^ within V1, the former acting in conjunction with L494I in C5 and the latter with a D601N pseudoreversion, largely suppressed the functional defects, with Env function becoming less dependent on particular gp120-contact residues (Leu^593^, Trp^596^ and Lys^601^) within the DSR.

One mechanism of reversion involved the acquisition of L494I in C5 that led to slightly improved gp120-gp41 association and fusion function with Asp^601^, but a WT-like phenotype also required deletion of the glycan at Asn^136^ in V1 through T138N. Outgrowth by Asn^601^-containing clones suggested that the negative charge of Asp^601^ was not favoured in the long term. Cell-cell fusion and viral replication were also restored by a short ΔN^139^INN deletion in V1 operating in conjunction with a D601N pseudoreversion. In this case Env fusogenicity was maintained despite a more labile gp120-gp41 complex. The ΔN^139^INN/K601N phenotype was largely recapitulated by S144N/K601N, suggesting that loss of the Asn^142^ glycan is sufficient to restore fusion function when Asn^601^ is present in the DSR. Mutations designed to ablate the remaining PNGSs at positions 141, 156, 160 and 186 within V1V2, did not restore function to Asn^601^-containing Envs, indicating a specific functional linkage between the DSR and the Asn^136^ and Asn^142^ glycans in the context of AD8 Env. It should be noted that V1V2 exhibits high degree of diversity in sequence, length and numbers of PNGSs [34], [35], [36], [37], [38], [39], [40], [41], [42], [43], [44]. It is therefore likely that the functional linkages between particular V1V2 glycans and residues within the gp120-gp41 association site will vary in a strain-dependent manner.

The impacts of the V1 glycan changes on the gp120-gp41 association site were probed further by combining T138N, L494I and ΔN^139^INN with various DSR mutations: L593V and K601D, which disrupt gp120-gp41 association and fusion function, and W596L which inhibits fusion [27], [28]. The reverting mutations were found to change the dependence of fusion and association on these DSR residues. For example, T138N/L494I rendered subunit association and fusion function largely independent of Leu^593^ and Lys^601^, whereas less dependence on Lys^601^ was observed with ΔN^139^INN. In the case of W596L, T138N and ΔN^139^INN were sufficient to confer WT fusion levels, but this did not involve changes to the WT-like gp120-gp41 association phenotype of W596L. The fusion gains conferred to W596L may involve transduction of a receptor-induced activation signal from gp120 to gp41 through the association site in manner that is less dependent on Trp^596^. The earlier finding that W596L blocks sCD4-induced formation of the gp41 prehairpin intermediate indicates that Trp^596^ can play a role in receptor-triggered gp41 activation [29]. The generation of functional Env complexes with stable gp120-gp41 association following the combination of V1 glycan mutations with various L593V, W596L or K601D DSR mutations, may be mediated by a shifting of the points of intersubunit contact to conserved residues at alternative positions within the synapse [e.g. Gln^591^, Gly^597^, Thr^606^ and Trp^610^ [27], [28], [76]], and/or other regions of gp41 that appear to interact with gp120, including the fusion-peptide proximal segment [77], HR1 [78], [79], and HR2 [76].

**Figure 8:**
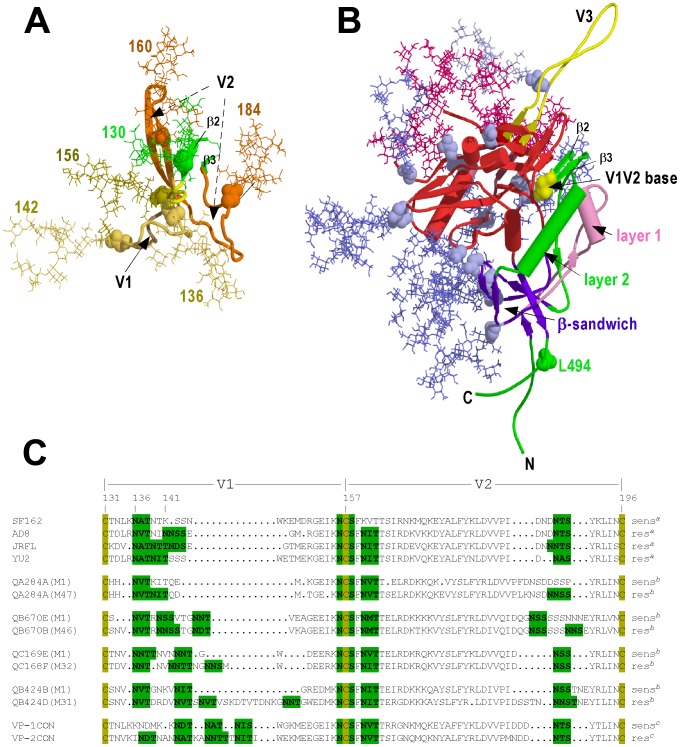
Structural models. ***A***, Homology model of oligomannose-glycosylated AD8 V1V2 based on the crystal structure of CAP45 V1V2 [7]. The V1V2 model, generated using the Modeller algorithm [113] within Discovery Studio 3.0, was glycosylated *in silico* with oligomannose side chains using the glycosciences.de server (http://www.glycosciences.de/modeling/glyprot/php/main.php) [114], [115]. The β2–β3 hairpin that forms the V1V2 base is colored green, V1 in yellow, V2 in orange. PNGSs (Asn residues shown in CPK) and oligomannose side chains are colored according to the V1V2 subdomain to which they are attached. ***B***, Model of oligomannose-glycosylated gp120 monomer. The model, prepared with the UCSF Chimera package [117], is based on the crystal structures of the complex formed between HXBc2 gp120 with gp41-interactive region (gp120 residues 31–284, 334–501), sCD4 and 48 d Fab (PDB ID 3JWD) and the YU2 gp120 (residues 285–333)-418d Fab-sCD4 complex (PDB ID 2QAD) [5], [8]. Oligomannose addition was performed *in silico* as for ***A***. The gp41 association site formed by the N- and C-terminal segments is colored green, the 7 stranded β-sandwich in purple, layer 2 and the V1V2 β2–β3 hairpin base in green, layer 1 in pink, the outer domain in red. Asn residues representing PNGSs are shown in CPK. Oligomannose glycans implicated in 2G12 recognition are colored crimson. The homology models were drawn using Pymol. ***C***, Alignment of V1V2 amino acid sequences. Selected V1V2 sequences were initially aligned using clustalx and then adjusted manually. PNGSs are highlighted in green. Residue numbering is according to HXB2. sens: neutralization-sensitive; res: neutralization-resistant. *^a^*, Neutralization susceptibility of Envs derived from subtype B HIV-1 reference strains as determined using macaque antisera raised to SF162 gp140 immunogen [36]; *^b^*, Neutralization susceptibility phenotype associated with primary (M1) and chronic (M47, M46, M32 and M31) phase subtype A V1V2 sequences as determined with autologous plasma [42]; *^c^*, Consensus V1V2 sequences derived from neutralization-sensitive (VP-1CON) and neutralization-resistant (VP-2CON) isolates obtained from a patient with cross-reactive neutralizing activity as determined with autologous plasma [44].

V1V2 comprises a conserved 4-stranded β-sheet minidomain that is stabilized by 2 interstrand disulfides (Cys^126^-Cys^196^ and Cys^131^-Cys^157^). The highly variable segments and PNGSs are for the most part contained within the connecting loops (**Fig. 8A**) [7]. A model of glycosylated AD8 V1V2 based on the CAP45 V1V2 structure [7] indicates that 6 N-linked glycans would encompass this minidomain in a hydrophilic shell (**Fig. 8A**). The most obvious changes due to T138N and ΔN^139^INN would be the loss of hydrophilic glycan bulk (∼1840 Å^3^ per high-mannose glycan; see [80]) and, for the latter, a shortening of the V1 loop (**Fig. 8A**). N-linked glycans have been found to induce local order in the backbone of protein loop regions by destabilizing the unfolded state, the increased entropy of the flexible glycan side chain compensating for the decreased entropy of the protein backbone [81], [82], [83]. The loss of the Asn^136^ and Asn^142^ glycans may lead to localized disorder and structural change and/or instability within V1, which in turn affects other structural elements of the gp120-gp41 complex. Cryoelectron tomography indicates that the Asn^136^ and Asn^142^ V1 glycans would be located at the apex of the trimeric Env spike whereas the gp120-gp41 association synapse is underneath the gp120 trimer [8], [12]. An allosteric mechanism whereby changes in V1 can affect the structure of the distal gp120-gp41 association site is suggested by the architecture of gp120, wherein 3 structurally plastic layers of the core domain are linked to the N- and C-terminal gp41-associating segments via an invariant 7-stranded β-sandwich [8] (**Fig. 8B**). Layer 2 connects the β2-β3 hairpin that forms the base of V1V2 via the β-sandwich to the gp120 N- and C-terminal segments (**Fig. 8A, B**). Thus changes in V1V2 structure and/or stability due to the loss of the Asn^136^ or Asn^142^ glycans may cause a distortion or displacement of layer 2, which is translated to the N- and C-terminal gp41-interacting segments via the β-sandwich. Our data suggest that the glycan changes in V1 observed here involve a structural remodeling of the gp120-gp41 association interface such that Env function is less dependent on particular gp120-contact residues within the DSR with conserved residues at alternative positions within the synapse being employed for intersubunit contact **[27]**, **[28]**.

The V1V2 minidomain has been shown to modulate the accessibility and/or conformation of the CD4-binding site and V3 loop [38], [84], [85], [86], [87], the latter of which mediates chemokine receptor binding. Our data indicate that the sCD4-binding abilities of monomeric gp120 molecules bearing 2^nd^ and 3^rd^ site mutations in various combinations were not significantly different to the WT, suggesting that changes in CD4-binding ability per se do not contribute to the mechanism of reversion. However, ΔN^139^INN/K601N-mediated cell-cell fusion and virus infectivity were more resistant to sCD4 inhibition suggesting that the conformation of the ΔN^139^INN/K601N Env trimer is subtly different to that of WT and T138N/L494I/K601N. sCD4 has been shown to primarily inhibit HIV-1 infection by inducing a transiently activated glycoprotein complex that rapidly undergoes irreversible conformational changes linked to a loss of function [88]. This sCD4-mediated inactivation correlates with a rapid decay in exposure of the HR1 coiled coil groove of the fusion-activated gp41 prehairpin intermediate. It may be that the apparently looser association between gp120 and gp41 of ΔN^139^INN/K601N alters the conformational pathway of the sCD4-induced state to enable maintenance of fusion competence.

A striking feature of gp120-gp41 is the occlusion of the conserved protein surface by a glycan shield comprising ∼24 N-linked glycans on gp120 and 4–5 on gp41 [3], [56], [89], [90], [91]. An evolving glycan shield is an important mediator of viral escape from NAbs where an increase in the number and/or a change in the position of PNGSs alter NAb sensitivity [35]. A number of studies have indicated that V1V2 plays a particularly important role in regulating neutralization resistance [36], [38], [41], [42], which generally correlates with V1V2 elongation and insertion of PNGSs, in some cases at and C-terminal to position 136 (**Fig. 8C**) [34], [37], [38], [39], [40], [42], [43], [44]. Our studies indicate that subtle changes to the glycan shield in V1 impact on neutralization by glycan-dependent brNAbs. For example, the Asn^136^ and Asn^142^ glycan mutations were found to increase the sensitivity of HIV-1 pseudovirions to the 2G12 NAb, which is dependent on high-mannose glycans on the outer face of gp120, including those attached to Asn^295^ and Asn^332^ at the base of V3, as well as Asn^339^, Asn^386^ and Asn^392^ [63], [64], [65], [66], [67], [68] (**Fig. 8B**). Neutralization epitopes within V3 appear particularly sensitive to changes in V1V2, which is likely due to the proximity between these variable structures in the context of trimeric gp120-gp41 [10], [12], [38], [84], [86]. The Asn^136^ and Asn^142^ V1 glycan deletions may modulate the structure and/or accessibility the 2G12 glycan epitope by altering V1V2-V3 interactions in the context of trimeric gp120. Alternatively, the enhanced neutralization of T138N and ΔN^139^INN by 2G12 may be a result of changes in the global antigenic structure of gp120-gp41, as was suggested by the finding that the V1 glycan deletions slightly enhanced the neutralization potency of polyclonal HIVIG. That V1V2 can regulate global antigenic structure via changes in the glycan shield has been suggested by the finding that glycosylation changes within gp120 at 197, 234, 295 and 301 contribute to the restoration of infectivity to viruses from which the entire V1V2 region had been deleted [92]. We also observed that ΔN^139^INN led to increased sensitivity to neutralization by NAb PG16, whose complex epitope includes the V1V2 glycans at Asn^156^ and Asn^160^ and is influenced by residues in V3 [7], [69], [70]. The AD8 V1V2 model suggests that the Asn^142^ glycan is proximal to the Asn^156^ glycan, implying that the N^139^INN deletion may relieve a steric constraint that enables better epitope access for PG16. Alternatively, or additionally, changes in the disposition of V3, as was implied by the increased sensitivity of ΔN^139^INN to neutralization by 2G12, may contribute to the increased neutralization efficacy of PG16. Overall, our data indicate that the Asn^136^ and Asn^142^ glycans of V1 can modulate local (PG16) and remote (2G12) glycan-dependent neutralization epitopes as well as the global antigenic structure of Env (HIVIG) and that these changes are functionally linked to a remodelling of the gp120-gp41 association site. Our data also imply that glycan shield evolution may indirectly affect the inhibitory potential of novel fusion blockers, such as PF-68742, for which the DSR is a component of their targeting mechanism [33]. Conversely, DSR sequence evolution driven by potential DSR-directed entry inhibitors may be associated with compensatory V1 glycan changes as described here and the coevolution of neutralization sensitivity.

Our previous work indicated that the gp120-gp41 association interface is structurally and functionally plastic despite exhibiting a high degree of sequence conservation [28]. In this study, we have found that changes at the 136 and 142 V1 glycans that are associated with neutralization sensitivity appear to remodel the gp120-gp41 association site, rendering certain highly conserved gp120-contact residues (i.e. Leu^593^, Trp^596^ and Lys^601^) less important for gp120 association and fusion function, and thereby implying that gp120-gp41 contact residues at alternative positions within the synapse become utilized for these functions. The allosteric modulation of the conserved DSR-C1–C5 synapse by distal V1 glycans may represent a mechanism whereby functionally relevant gp120-gp41 association is maintained as the virus acquires neutralization resistance due to the evolution of its glycan shield.

## Materials and Methods

### Env expression vectors and proviral clones

The preparation of the cytomegalovirus promoter-driven HIV-1_AD8_ Env expression vector, pCDNA3.1-AD8*env*, is described elsewhere [28]. pΔKAD8*env* was derived by religation of the end-filled *Hind*III and *Eco*RI sites of pCDNA3.1-AD8*env*. *In vitro* mutagenesis of the gp41 region was carried out by overlap extension PCR. Mutants of the pAD8 infectious clone (obtained from K. Peden [93] were prepared by transferring the *Eco*RI-*Bsp*MI *env*-containing fragment from pCDNA3.1-AD8*env* vectors into pAD8. Bacteriophage T7 promoter-driven gp120 expression vectors, based on pTM.1 [94], were generated by ligating PCR-amplified HIV-1_AD8_ gp120 fragments into the *Nde*I and *Stu*I sites of pTMenv.2 [95] to give pTM-AD8*gp120*.

### Infection of PBMCs

PBMC infections were conducted as described previously [96]. Briefly, PBMCs isolated from buffy packs (Red Cross Blood Bank, Melbourne) were stimulated with phytohemagglutinin (10 µg/ml; Murex Diagnostics) for 3 days in RPMI 1640 medium containing 10% fetal calf serum and interleukin-2 (10 units/ml; Boehringer-Mannheim). Virus stocks were prepared by transfecting 293T cell monolayers with pAD8 infectious clones using Fugene 6 (Roche). Virus-containing transfection supernatants were normalized according to reverse transcriptase (RT) activity, and then used to infect 10^5^ PBMCs in a 96-well tissue culture plate (eight 10-fold serial dilutions of each virus were tested in triplicate). The supernatants were assayed for RT activity at various time points.

### Sequential passage of cell-free K601D virus in PBMC

Phytohemagglutinin-stimulated PBMCs were infected with equivalent amounts of wild type (WT) and K601D-mutated HIV-1_AD8_ (according to RT activity) in parallel and maintained in culture for 10 days. Cell-free culture supernatants were filtered (0.45 µm pore size) and normalized according to RT activity prior to the next passage (5 passages in total). Genomic DNA was extracted from infected PBMCs using Qiagen DNeasy. The viral DNA fragment encompassed by nucleotides 5954–9096 (HXB2R numbering convention) was PCR-amplified using Expand HiFi (Roche) and the primers, 5′-GGCTTAGGCATCTCCTATGGCAGGAAGAA (Env1A) and 5′-TAGCCCTTCCAGTCCCCCCTTTTCTTTTA (Env1M) [97]. The amplified sequences were ligated into pGEM-T or pΔKAD8*env* (*Kpn*I-*Xba*I) and the entire *env* open reading frame sequenced using ABI BigDye terminator 3.1.

### Western blotting

Lysates of Env-expressing 293T cells or virions pelleted from pAD8-transfected 293T cell supernatants were subjected to SDS-PAGE under reducing conditions, transferred to nitrocellulose and then probed with mAb C8 to gp41 (from G. Lewis [98], DV-012 to gp120 (from M. Phelan [99], [100], or mAb 183 to CA (from B. Chesebro and K. Wehrly [101], [102] (AIDS Research and Reference Reagent Program, NIAID) as described [28].

### Luciferase reporter assay of cell-cell fusion

Cell-cell fusion assays were conducted as described [77]. Briefly, 293T effector cells were cotransfected with pCDNA3.1-AD8*env* or pΔKAD8*env* and pCAG-T7 [103] plasmids, while BHK21 target cells were cotransfected with pT4*luc* [27] and pc.CCR5 (AIDS Research and Reference Reagent Program from N. Landau [104] or a panel of CCR5 mutants in the pcDNA3 expression vector (kind gifts of J. S. Sodroski and R. W. Doms [105], [106]). The Y14N mutation was introduced to pc.CCR5 using the Quikchange II XL kit (Stratagene). At 24 h posttransfection, targets and effectors were cocultured in triplicate in a 96-well plate (18 h, 37°C) and then assayed for luciferase activity (Promega SteadyGlo, Madison, Wis.). The sensitivities of WT and mutant Env proteins to the fusion inhibitor peptide C34 (WMEWDREINNYTSLIHSLIEESQNQQEKNEQELL; Mimotopes, Australia [62]) were determined by coculturing effector and target cells in the presence of serially diluted C34. The sensitivities of WT and mutant Env proteins to sCD4 (NIH AIDS Research and Reference Reagent Program) were determined by incubating the Env-expressing 293T cells with a dilution series of sCD4 for 3.5 h followed by coculturing the effector and target cells in the presence of sCD4 for 8 h.

### Single cycle infectivity assays

Single cycle infectivity assays were conducted as described [77]. Briefly, Env-pseudotyped luciferase reporter viruses were produced by cotransfecting 293T cells with pCDNA3.1-AD8*env* or pΔKAD8*env* vectors plus the luciferase reporter virus vector, pNL4.3.Luc.R^−^E^−^ (AIDS Research and Reference Reagent Program, N. Landau [107]), using Fugene 6. The infectivity of pseudotyped viruses was determined in U87.CD4.CCR5 cells (AIDS Research and Reference Reagent Program, H. Deng and D. Littman [108]).

### Biosynthetic labelling and immunoprecipitation

293T cells were transfected with pCDNA3.1-AD8*env* or pΔKAD8*env* vectors. At 24-h posttransfection, the cells were incubated for 30 min in cysteine and methionine-deficient medium (MP Biomedicals, Seven Hills, NSW, Australia), and then labelled for 45 min with 150 µCi Tran-^35^S-label (MP Biomedicals). The cells were then washed and chased in complete medium for 5–6 h prior to lysis. Cell lysates and clarified culture supernatants were immunoprecipitated with IgG14 or HIVIG and protein G Sepharose and subjected to SDS-PAGE in the presence of ß-mercaptoethanol [28]. The labelled proteins were visualized by scanning in a Fuji phosphorimager. Quantitation of bands was performed using Image Gauge (FUJIFILM) software.

### Kifunensine treatment of transfected cells

To determine the effects of the competitive α-mannosidase inhibitor, kifunensine (Sigma), on Env synthesis and processing, cell-cell fusion and gp120-gp41 association, the compound was added to 293T cells at the time of transfection, and was maintained at the concentrations indicated in the results section through each step of the assays described above.

### gp120-sCD4 binding assay

293T cells were cotransfected with pTM-AD8*gp120* and pCAG-T7 vectors using Fugene 6. At 24-h posttransfection, the cells were incubated for 30 min in cysteine and methionine-deficient medium, labelled for 45 min with 150 µCi Tran-^35^S-label, and then washed and chased in complete medium for 6 h. The clarified culture supernatants were adjusted to 0.6 M KCl, 1 mM EDTA and 1% w/v Triton-X100 and the gp120 content quantified following immunoprecipitation with IgG14 and protein G Sepharose, reducing SDS-PAGE and scanning in a Fuji phosphorimager. Equivalent amounts of WT and mutant gp120 proteins were incubated with serial dilutions of sCD4 (1 h, room temperature) and then incubated with mAb OKT4 and BSA-Sepharose on a vibrating platform (30 min, room temperature). After pelleting the BSA-Sepharose, protein complexes were coprecipitated using protein G-Sepharose and gp120 quantified following reducing SDS-PAGE and scanning in a Fuji phosphorimager.

### Neutralization of infection by sCD4-IgG2

Wild type, T138N/L494I/K601N and ΔN^139^INN/K601N virus stocks were adjusted such that ∼2×10^6^ relative light units (RLU) were obtained following a 48 h-infection of TZM-bl cells, a HeLa cell line expressing CD4 and CCR5 and harbouring integrated copies of the luciferase and β-galactosidase genes under control of the HIV-1 promoter (obtained from J. C. Kappes, X. Wu and Tranzyme Inc., NIH AIDS Research and Reference Reagent Program [109], [110], [111]). A dilution series of CD4-IgG2, a tetrameric fusion protein comprising human IgG in which the Fv domains have been replaced by domains 1 and 2 of human CD4 (Progenics Pharamceuticals, NIH AIDS Research and Reference Reagent Program), was incubated with the viruses for 4 h at 37°C prior to addition to TZM-bl cells (200 µl; 10^4^ cells in 96-well culture plates). The cells were lysed 48 later and assayed for luciferase activity (Promega).

### Neutralization assay

Neutralization assays were conducted using a modification of the method of Dhillon et al. [112]. Briefly, a solution of Env-pseudotyped luciferase reporter viruses determined previously to give ∼5×10^5^ RLU following infection of U87.CD4.CCR5 cells was mixed with an equal volume of serially diluted IgG and incubated for 1 h at 37°C. One hundred microliters of the virus-IgG mixtures was then added to U87.CD4.CCR5 cells (10^4^ cells per well of a 96-well tissue culture plate, 100 microlitres) and incubated for 2 days prior to lysis and assay for luciferase activity (Promega, Madison, WI). Neutralizing activities for antibody samples were measured in triplicate and reported as the average percent luciferase activity. Purified IgG of monoclonal NAbs 2F5, 2G12 and IgGb12 were obtained from Polymun Scientific (Austria), PG16 was obtained from the International AIDS Vaccine Initiative, while HIVIG was obtained from F. Prince through the NIAID AIDS Research and Reference Reagent Program.

### Molecular modeling

A homology-based model of HIV-1_AD8_ V1V2 was generated from PDB entry 3U4E [7] using the Modeller algorithm [113] within Discovery Studio, version 3.0 (Accelrys). Five independent models were generated from iterative cycles of conjugate-gradient energy minimization against spatial constraints derived from the template crystal structure. The model with the lowest energy (probability density function) was glycosylated *in silico* with oligomannose side chains using the glycosciences.de server (http://www.glycosciences.de/modeling/glyprot/php/main.php) [114], [115].

## Supporting Information

Figure S1 Effect of kifunensine on Env synthesis and function. ***A***, Western blot. Kifunensine was added to 293T cells at the time of transfection with ΔKAD*env* expression vectors. The transfected cells were cultured in the presence and absence of kifunensine for a further 48 h prior to lysis, reducing SDS-PAGE and Western blotting with DV-012 to gp120 (top panel) or 2F5 to gp41 (bottom panel). ***B***, Cell-cell fusion. 293T cells were cotransfected with ΔKAD*env* expression vectors plus pCAG-T7 and cultured in the presence or absence of 10 µM kifunensine. At 24 h posttransfection, the cells were detached and cocultured for a further 18 h with pT4*luc* plus pcCCR5-cotransfected BHK-21 cells in the presence or absence of 10 µM kifunensine prior to lysis and assay for luciferase activity. ** *P*<0.01; **, *P*<0.001; T138N/L494I/K601N and ΔNINN/K601N *versus* K601D at the same kifunensine concentration; 2-tailed unpaired t test assuming unequal variances (n = 5). ***C***, gp120-gp41 association. Kifunensine was added to 293T cells at the time of transfection with ΔKAD*env* expression vectors. At 24 h posttransfection, the cells were pulse-chase metabolically labelled in the presence or absence of 10 µM kifunensine prior to immunoprecipitation of culture supernatants (s) and cell lysates (c) with HIVIG, reducing SDS-PAGE and phosphorimager scanning.
(TIF)

Table S1 Summary of cell-cell fusion, gp120-gp41 association and replication characteristics of revertant Env clones.
(DOCX)
