# Supplementary figures and images for: Allosteric Modulation of the HIV-1 gp120-gp41 Association Site by Adjacent gp120 Variable Region 1 (V1) N-Glycans Linked to Neutralization Sensitivity
Source: PLoS Pathog. 2013 Apr 4;9(4):e1003218. doi: 10.1371/journal.ppat.1003218 (PMC3616969; doi:10.1371/journal.ppat.1003218)

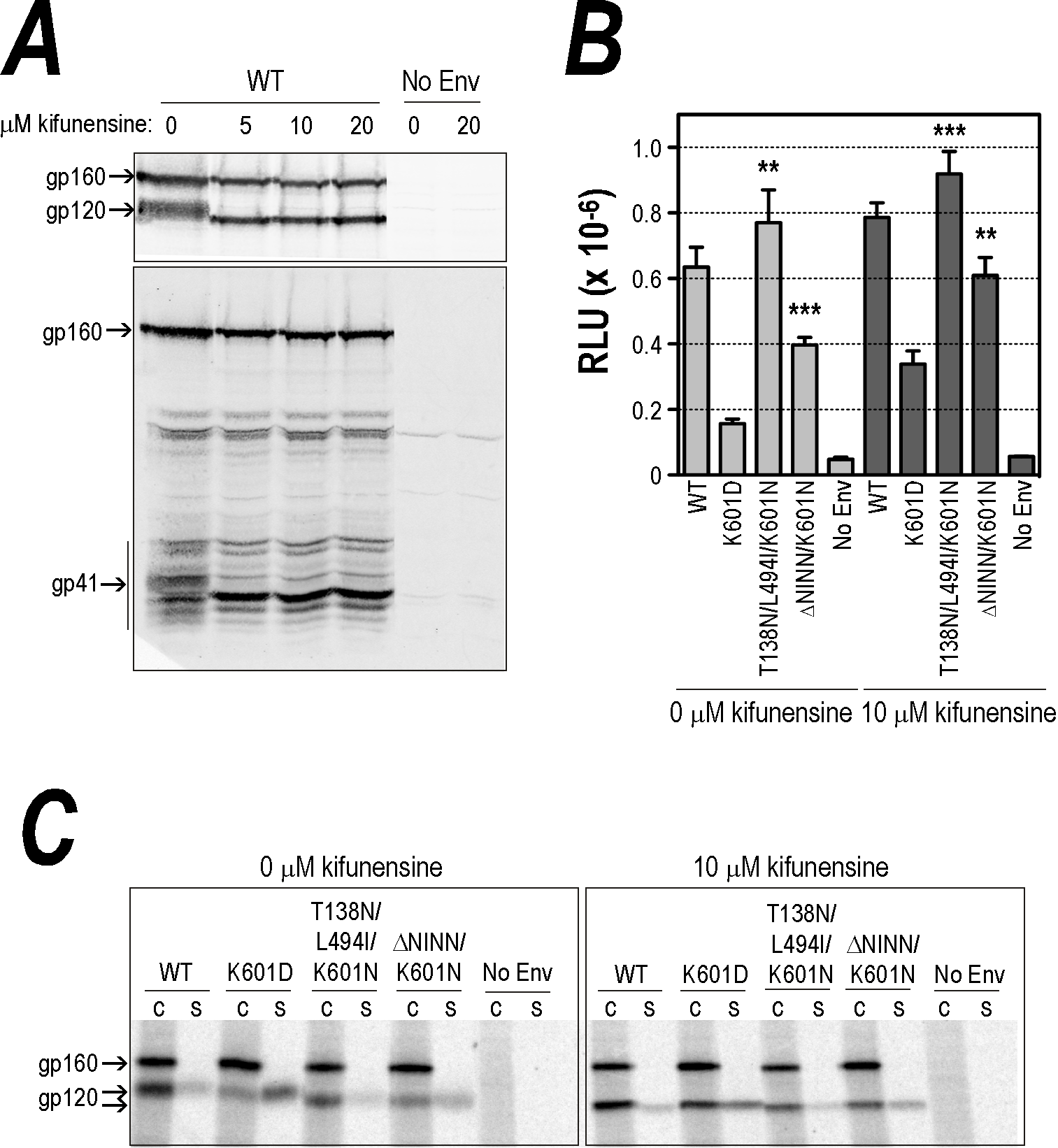

Supplement: Figure S1 — Effect of kifunensine on Env synthesis and function. A, Western blot. Kifunensine was added to 293T cells at the time of transfection with ΔKADenv expression vectors. The transfected cells were cultured in the presence and absence of kifunensine for a further 48 h prior to lysis, reducing SDS-PAGE and Western blotting with DV-012 to gp120 (top panel) or 2F5 to gp41 (bottom panel). B, Cell-cell fusion. 293T cells were cotransfected with ΔKADenv expression vectors plus pCAG-T7 and cultured in the presence or absence of 10 µM kifunensine. At 24 h posttransfection, the cells were detached and cocultured for a further 18 h with pT4luc plus pcCCR5-cotransfected BHK-21 cells in the presence or absence of 10 µM kifunensine prior to lysis and assay for luciferase activity. ** P<0.01; **, P<0.001; T138N/L494I/K601N and ΔNINN/K601N versus K601D at the same kifunensine concentration; 2-tailed unpaired t test assuming unequal variances (n = 5). C, gp120-gp41 association. Kifunensine was added to 293T cells at the time of transfection with ΔKADenv expression vectors. At 24 h posttransfection, the cells were pulse-chase metabolically labelled in the presence or absence of 10 µM kifunensine prior to immunoprecipitation of culture supernatants (s) and cell lysates (c) with HIVIG, reducing SDS-PAGE and phosphorimager scanning. (TIF) [file ppat.1003218.s001.tif]
