# Supplementary material for: Allosteric Modulation of the HIV-1 gp120-gp41 Association Site by Adjacent gp120 Variable Region 1 (V1) N-Glycans Linked to Neutralization Sensitivity
Source: PLoS Pathog. 2013 Apr 4;9(4):e1003218. doi: 10.1371/journal.ppat.1003218 (PMC3616969; doi:10.1371/journal.ppat.1003218)
Supplement: Table S1 — Summary of cell-cell fusion, gp120-gp41 association and replication characteristics of revertant Env clones. (DOCX) [file ppat.1003218.s002.docx]

Table S1. Summary of cell-cell fusion, gp120-gp41 association and replication characteristics of revertant Env clones.

Relative fusion function*^a^* gp120 shedding index*^b^* Replication*^c^*

WT 100 1 ++++

K601D 9.8 6.8 -

K601N 15.2 4.4 +/-*^d^*

L494I/K601D 32.9 3.4 +/-

T138N/K601D 5.8 2.7 nd^e^

T138N/L494I/K601D 98.6 1.4 +++

T138N/L494I/K601N 103.5 1.0 +++

ΔNINN/K601D 72.3 4.5 -

ΔNINN/K601N 75.2 2.7 +++

*^a^*Data from FIG. 3D. The background RLU obtained with pcDNA3.1-transfected effector cells was subtracted from the RLUs obtained with Env expression vector-transfected effector cells and normalized against the fusion activity obtained with WT.

*^b^*Data from FIG. 3C.

*^c^*Data from FIGS. 3A and B.

*^d^*+/- denotes a low level of replication in PBMCs obtained from donor A but not from donor B (FIGS. 3A and B, respectively).

*^e^*nd, not determined
